# Supplementary material for: Comparative Short- and Long-Term Effectiveness and Safety of Pramipexole and Aripiprazole Augmentation in Treatment-Resistant Unipolar Depression: An Observational Study
Source: Biomedicines. 2024 Sep 10;12(9):2064. doi: 10.3390/biomedicines12092064 (PMC11428619; doi:10.3390/biomedicines12092064)
Supplement: Supplementary file 1 [file biomedicines-12-02064-s001.zip › biomedicines-3088404-supplementary.pdf]

Supplementary materials

ESM Table S1. A)-C) Odds ratios of remission, response and improvement at 24 vs. 12 weeks for pramipexole vs. aripiprazole. D) Regression coefficients of functioning over time by type of augmentation. Results obtained from repeated measures generalized linear mixed models.

A) Remission (HDRS ≤ 7)

	**OR (95%CI)**	**p-value**
Intercept	0.18 (0.06; 0.54)	0.002
**Type of augmentation**		
aripiprazole	r.c.	
pramipexole	4.24 (1.54; 11.67)	**0.005**
**Time**		
12-week	r.c.	
24-week	1.67 (0.67; 4.15)	0.265
**Augmentation*time**	1.61 (0.53; 4.94)	0.403
**Propensity score**	1.79 (0.57; 5.60)	0.314

Binomial distribution with a logit-link function.

OR: Odds ratio; r.c.: reference category;

95% CI: 95% Confidence Interval

B) Response (≥50% reduction in HDRS)

	**OR (95%CI)**	**p-value**
Intercept	0.43 (0.16; 1.12)	0.085
**Type of augmentation**		
aripiprazole	r.c.	
pramipexole	3.77 (1.48; 9.60)	**0.006**

**Time**		
12-week	r.c.	
24-week	1.30 (0.57; 2.96)	0.539
**Augmentation*time**	1.39 (0.47; 4.06)	0.550
**Propensity score**	1.30 (0.44; 3.83)	0.633

Binomial distribution with a logit-link function.

OR: Odds ratio; r.c.: reference category;

95% CI: 95% Confidence Interval

C) Improvement (CGI-i < 2)

	**OR (95%CI)**	**p-value**
Intercept	1.79 (0.64; 5.02)	0.268
**Type of augmentation**		
aripiprazole	r.c.	
pramipexole	2.76 (0.94; 8.10)	0.064
**Time**		
12-week	r.c.	
24-week	0.81 (0.33; 2.01)	0.648
**Augmentation*time**	0.94 (0.27; 3.26)	0.927
**Propensity score**	2.34 (0.69; 7.91)	0.172

Binomial distribution with a logit-link function.

OR: Odds ratio; r.c.: reference category;

95% CI: 95% Confidence Interval

D) GAF

	b (95%CI)	p-value
Intercept	3.87 (3.81; 3.94)	**<0.001**
**Type of augmentation**		
Aripiprazole	r.c.	
Pramipexole	0.09 (0.03; 0.15)	**0.003**
**Time**		
Baseline	r.c.	
12-week	0.16 (0.09; 0.22)	**<0.001**
24-week	0.19 (0.12; 0.25)	**<0.001**
**Augmentation*time**		
aripiprazole*12-week	0.10 (0.02; 0.19)	**0.014**
aripiprazole*24-week	0.13 (0.04; 0.21)	**0.004**
**Propensity score**	0.13 (0.06; 0.20)	**<0.001**

Gamma distribution with a log-link function.

b: beta coefficient; r.c.: reference category;

95% CI: 95% Confidence Interval

ESM Table S2. Predicted rates of remission, response, improvement and predicted mean of GAF score according to type of augmentation and time, adjusted for the propensity score. Results obtained from repeated measures generalized linear mixed models.

	Baseline	12-week	24-week
**Remission, estimated rate (*95% CI*)**			
Pramipexole		49.7% (*37.7% - 60.9%*)	72.7% (*61.8% - 82.0%*)
Aripiprazole		18.9% (*9.6% - 33.7%*)	28.1% (*16.0% - 44.3%*)
**Response, estimated rate (*95% CI*)**			
Pramipexole		64.1% (*51.9% - 74.8%*)	76.2% (*64.8% - 84.8%*)
Aripiprazole		32.2% (*19.3% - 48.5%*)	38.0% (*23.9% - 54.5%*)
**Improvement, estimated rate (*95% CI*)**			
Pramipexole		87.2% (*77.8% - 93.0%*)	83.9% (*73.6% - 90.7%*)
Aripiprazole		71.2% (*54.3% - 83.8%*)	66.7% (*49.4% - 80.4%*)
**GAF, estimated mean (*95% CI*)**			
Pramipexole	55.5 (*53.6 - 57.4*)	71.9 (*68.9 - 75.1*)	75.7 (*72.5 - 79.1*)
Aripiprazole	50.6 (*48.3 - 52.9*)	59.1 (*55.9 - 62.4*)	60.8 (*57.4 - 64.4*)
